# Living in Liquid Times: The Relationships among Job Insecurity, Life Uncertainty, and Psychosocial Well-Being

**DOI:** 10.3390/ijerph192215225

**Published:** 2022-11-18

**Authors:** Antonio Chirumbolo, Antonino Callea, Flavio Urbini

**Affiliations:** 1 Department of Psychology, Sapienza University of Rome, 00185 Rome, Italy; 2 Department of Humanities, LUMSA University, 00193 Rome, Italy

**Keywords:** job insecurity, life uncertainty, psychosocial well-being, COVID-19 pandemic

## Abstract

Stress research has widely documented how uncertainty represents a strong stressor that, in general, is negatively associated with well-being. While the literature on job insecurity about this topic is extensive and exhaustive, empirical research on the outcomes of life uncertainty, namely the perception and feeling of precariousness regarding the present and future of one’s own life, is yet to be fully explored. In the present paper, we aimed to investigate the relationships among job insecurity, life uncertainty, and psychosocial well-being outcomes, specifically, with a focus on job satisfaction and burnout. The participants were 357 workers (M = 146 and F = 211), with an average age of 41.78 y.o. (SD = 13.49), who completed an online questionnaire containing, in addition to sociodemographics information, measures of the study variables, namely job insecurity, life uncertainty, job satisfaction, and burnout. The results pointed out negative relationships of both job insecurity and life uncertainty with individual well-being, as they were negatively associated with job satisfaction and positively related to burnout. In a path analysis with latent variables, life uncertainty proved to fully mediate the relationship between job insecurity and psychosocial well-being.

## 1. Introduction

Many scholars from various disciplines have stressed that contemporary society is characterized by high levels of uncertainty, so much so, that it has often been called the age of chaos [1], the age of uncertainty [2], or the age of anxiety and fear [3]. These feelings of uncertainty and insecurity are felt by individuals in almost every area of daily life. When interviewed in a survey panel conducted by the EU with random samples, in 2020 and 2021, participants from various European countries reported how the most frequently experienced emotional states were “uncertainty” at first, followed by “helplessness” as the third recurrent option [4,5]. Moreover, regarding economic and financial perceptions, at the end of the first quarter of 2021, about 31% of European citizens saw their personal financial situation worsen during the pandemic and a further 26% expected this negative appraisal to still happen soon [5]. In the same vein, during the pandemic, psychological maladaptive symptoms pervasively increased all over the world, which has been well documented in the scientific literature [6,7,8]. This general state of uncertainty, financial concerns, and generalized personal and psychological discomfort, have been found to be especially true concerning the world of work [9,10,11,12].

### 1.1. Job Market Outcomes of the Pandemic

In general, the consequences on job markets due to the pandemic period have been well documented by many studies [13] that have reported comprehensive influences on both the number of hours of work and job losses [14]. Increased unemployment rates were somewhat pushed by pandemic measures such as lockdowns and social distancing [15,16,17]. However, since the impact of COVID-19 was unequal among workers of different ages, unemployment was also probably driven by a growing presence of “discouraged workers”, that is, workers who had stopped actively searching for jobs, and in this way, practically withdrew from the labor force [15]. The distribution of costs in terms of job and income losses was unbalanced [18], as there was more of an influence on financially vulnerable populations [19]. In the same vein, workers with lower levels of education, younger adults, and immigrants were also less likely to perform their work tasks from home, and therefore, were more likely to lose their jobs [20]. While the decreases in job opportunities were somehow uniform across industries and occupations, with the exception for those in front line medical-related jobs [14], small businesses were apparently more negatively affected as compared with major industries [21].

In Italy, the pandemic emergency has had direct repercussions, firstly, on the social system, and then, on the economic system [22]. The lockdown policies resulted in the suspension of many work and commercial activities with consequences on the entire work sector with similar outcomes as previously reviewed [22]. However, in the Italian socioeconomic context, the level of labor market insecurity was already among the highest in Europe, being lower only than Greece, Spain, and Turkey [23] This tendency also consistently occurred during the last pandemic years [5]. The Italian labor market suffered by using explicit legislative interventions, due to both economic crises and national laws, which were characteristic of both the public and private sectors [24]. Indeed, some legislative interventions froze the growth of public-sector wages (see Law n.122/2010 and Law n.122/2013). Furthermore, legislative interventions were made to reduce guarantees and protections, both for permanent and temporary employees [25]. For example, Law n.183/2014 abolished many regulations and protections for permanent workers.

As a result of the social and economic impact of the COVID19 pandemic [13], uncertainty and job insecurity increased pervasively in different layers of the population, with millions of people worldwide losing their jobs [26]. Today, the nature of work has changed as compared with the past decades, i.e., from safe and guaranteed jobs to structurally precarious and insecure jobs [5]. As occupational risks have increased, such as becoming unemployed or having a temporary contract, the perceptions and fear of job insecurity among employees have increased. Many authors have questioned whether job insecurity could be a challenge or a hindrance to employees, and have concluded that it negatively affects employees lives [27,28]. Consequently, job insecurity currently represents one of the most powerful stressors at work with several consequences both on individuals and on organizations themselves [29,30,31].

### 1.2. The Theoretical Framework: Uncertainty as a Source of Stress

Many scholars have suggested that uncertainty is, for most individuals, a powerful source of stress [30,31,32,33]. The transactional theory of stress by Lazarus and Folkman [33,34,35,36,37,38,39] posits that stress is an outcome that stems from a transaction between an individual and the environment, a process which entails perceptions, expectations, interpretations, and coping responses of an individual [33]. Stress reactions can occur at different levels and can be affective, cognitive, or behavioral [33,34,35,36,37,38,39]. In this theoretical framework, reactions to stress derive from an individual’s evaluation of a situation, a process called appraisal. Individuals can appraise a situation as challenging or threatening, where a threat implies the perception of a risk of a negative event occurring in the future [33]. The experience of stress typically arises when an individual believes that the accessible strategies and resources are not adequate to face the negative aspects of the situation and, in general, a stressor is considered to occur when an individual appraises the presence of a threat in the environment [33]. According to the authors, there are environmental situations and events that “are treated as normatively stressful” [33], (p. 83), that is, some situations, more than others, have the formal properties that create the potential for threat and harm. These events/situations are generally considered and appraised to be more stressful by individuals [33]. In this line of reasoning, Lazarus and Folkman [33] identified novelty, (low) predictability, ambiguity and uncertainty, as relevant situational factors that would be more likely to be appraised as threatening and stressful, and thus, more likely to generate stress reactions. In particular, and within this theoretical framework [33,34,35,36,37,38,39], in the present paper, we focused on uncertainty as a prominent source of stress for individuals that would be capable of eliciting negative stress reactions. In this sense, Lazarus and Folkman [33] have clearly stated that it is the feeling of uncertainty per se, more than the event that a threat will be actually realized, that precisely constitutes a great source of stress for many people. In the same vein, Mischel [40] investigated the reaction to uncertainty in illness and explained that individuals actively built meaning regarding illness events, and uncertainty generally indicated the absence of meaning. According to Mischel [40], therefore, it was specifically this absence of meaning involved in uncertainty that constituted a strong factor for individual strain reactions.

However, not all individuals suffer from uncertainty in the same way. In fact, intolerance of uncertainty also constitutes an individual difference variable, since people vary according to their psychological tolerance towards environmental situations that are considered to be threatening and frightening because of their unpredictability [41]. In general, uncertainty is characterized by the perception that a negative event may or may not occur and that there is no conclusive way to predict such events [42]. Individuals who are intolerant of such uncertainty are more likely to interpret all ambiguous situations as threatening [43]. This interpretation generally contributes to significant somatic stress reactions [44,45]. Individual differences in intolerance of uncertainty have been found to be related to extreme worry [46] and anxiety state [44], and to have positive relationships with anxiety problems, such as generalized anxiety disorder, obsessive compulsive disorder, and panic disorder [47,48,49]. Furthermore, high intolerance of uncertainty appears to weaken problem-solving skills, leading to inaction, procrastination, indecision, and avoidance of ambiguous situations [50]. Overall, the findings suggest intolerance of uncertainty represents an important correlate across anxiety disorders and depression [51] with documented neurobiological outcomes [52].

Within this theoretical background, many authors have shown that people feel uncomfortable when they experience personal uncertainty [53] and have negative emotional responses when they face uncertainty [44], and that experimentally induced uncertainty increases both systolic and diastolic blood pressure when anticipating the occurrence of a possible threat of unknown intensity [45]. In addition, being uncertain about one’s health is a significant predictor of stress. In fact, perceived uncertainty about symptoms, treatment, and health outcomes have been found to be major predictors of stress, with uncertainty also exhibiting a mediating role between seriousness of illness and distress [40,54,55,56]. Furthermore, uncertainty has also been shown to affect negative emotions [57].

### 1.3. Job Insecurity and Psychosocial Well-Being

Uncertainty about one’s own job, namely job insecurity, refers to the subjective appraisal made by a worker about the probability and concern associated with the future involuntary loss of both a job position itself and important intrinsic characteristics of the job [29,31,32,58]. Currently, job insecurity is considered to be one of the most powerful sources of stress [29,30,31,32,58]. In fact, the perceived risk or fear of losing one’s job is perceived as a threat most of the times, which the individual interprets as negative, and thus, tries to cope and reduce [59]. As the term itself indicates, job insecurity implies a great deal of uncertainty to which people negatively react, as predicted by the transactional theory of stress [29,30,31,32,33,59]. Therefore, the phenomenon of job insecurity represents a threat either to the job itself or to valued aspects of the job, dealing with the realm of the subjective appraisal of an uncertain (i.e., insecure) and unwanted (i.e., involuntary) event (i.e., job loss) [29,30,31,58,59].

This definition aims to integrate various aspects that represent the constructs of job insecurity. A key role, in this case, is played by the subjective component underlying the evaluation of an uncertain event, namely the future unwanted loss of work, which the individual performs based on his own perceptions of the work environment and broader context (social, political, economic, and cultural). In this sense, research has shown that the same “objective” work situation can be perceived quite differently by people [42]. In fact, it is not certain that individuals who find themselves in the same work situation experience the same levels of job insecurity or that they are equally affected by it [59]. In this regard, it should be emphasized that, although it is strongly correlated, the subjective perception of job insecurity represents a phenomenon that is different from an “objective” job insecurity derived, for example, from the specific occupational status, as in the case of precarious or temporary employment [31].

Empirical research over the last 25 years has shown that the psychosocial costs of job insecurity are very high, and its outcomes are negative and harmful to the physical and psychological health of individuals and the well-being of organizations, as many meta-analyses have convincedly shown [30,31,60,61,62].

Individuals who perceive greater job insecurity have worse psychological health [63]: they feel more depressed, anxious, stressed, and have lower self-esteem and lower sense of self-efficacy [30,31]. Greater job insecurity is also associated with higher interpersonal conflict in different domains, both within the family [64,65,66] and at work with colleagues [67,68]. Furthermore, from an economic point of view, greater perception of job insecurity also means greater probability of giving up important personal life goals (e.g., starting a family, getting married, and taking out a mortgage) and less propensity to consume durable and non-durable goods [69,70], greater probability to have worse working conditions [71], and perhaps for this reason, to highlight a worse wage gap than individuals who feel subjectively confident in their work [72].

Job insecurity is also related to a significantly worse state of physical health [63]: individuals with higher insecurity, in fact, more frequently present greater somatic symptoms such as, for example, greater incidence of cardiovascular disorders (e.g., arterial hypertension, tachycardia, and cholesterol levels), diseases of the respiratory tract (cold, flu, sore throat, etc.), and pain in the musculoskeletal system (back pain, joint problems, etc.) (for reviews see [62,63,73,74]).

The negative associations of job insecurity with the well-being and functioning of organizations are not far behind. Workers with greater job insecurity are less satisfied with their jobs, less motivated, and less committed; they perform lower and exhibit less vigor, dedication, and enthusiasm at work [30,31]. Furthermore, workers with greater job insecurity appear to have a lower level of identification and trust towards their own organization, at the same time feeling much less connected and committed to it [75]. From the point of view of work behavior, workers with greater job insecurity are more susceptible to accidents and injuries at work [76,77] and show greater propensity to carry out persistent hindering and non-cooperative behaviors, such as chronic absenteeism and systematic delays at work, sometimes committing real acts of sabotage against a company [78,79,80]. Furthermore, insecure and precarious workers who have a more vulnerable employment status are more frequently subjected to sexual harassment and bullying within organizational contexts [81]. Importantly, these negative associations have been robustly documented both regarding the perception and fear of losing one’s job (quantitative job insecurity), and the perception and fear of losing important aspects of one’s job (qualitative job insecurity) [63].

In conclusion, several studies have highlighted that both objective and subjective job insecurities could be configured as real psychosocial risk factors [75], above all, when they become chronic; and therefore, create fractures between individuals, groups, and organizations, resulting in profound social and economic inequalities [72]. On the one hand, the main direct relationships between job insecurity and its outcomes appear to be well established; on the other hand, possible intervening and mediating variables of these relationships are still open to investigation and need further analysis [29,63].

### 1.4. Life Uncertainty

Life uncertainty has been defined as a subjective assessment “regarding the precariousness, uncertainty, and temporary nature of one’s present and future life” [69]. The feeling of existential precarity represents a commonly shared sense of vulnerability that characterizes everyone’s life [81]. Bauman [2] underlined how contemporary fragmented and atomized society inevitably increased the general mood of precariousness experienced everyday by individuals, nested in a personal narrative of biographical uncertainty [2]. In fact, European survey data have well documented that the most common emotional feeling in the last two years has been “uncertainty” [4], and that uncertainty accurately described and pervaded almost all aspects of the COVID-19 pandemic era we have just been living in [82,83]. Despite similarities in labels, related constructs have been dealing with a somewhat different phenomenon than life uncertainty as we intended it. For instance, personal uncertainty has been referred to as the uneasy feeling experienced when being uncertain about oneself [53]. Similarly, life precarity has been described as the feeling of precariousness nested in one’s position within the job market, with those with atypical, temporary, and unguaranteed insecure jobs feeling more precariousness [82,83,84]. Rather, in this paper, we refer to an individual’s perception that his/her present life is characterized by a sense of temporariness and instability, and that individual future life plans are surrounded by feelings of fragility and uncertainty. Although Lazarus and Folkman [33] did not explicitly mention it, according to the transactional theory of stress [33], feelings of uncertainty in life can be surely regarded as a potential source of stress, and thus, expected to elicit, on average, stress reactions similar to other uncertainty situations. Surprisingly, the research in this domain appears very scant and needs to be expanded. One study investigated the relationships between insecurity at work and life uncertainty in the realm of consumer behavior [69]. However, to the best of our knowledge, no other study has dealt with the potential negative reactions to life uncertainty in the domain of psychosocial well-being, nor has investigated its possible mediating role in the relationship between job insecurity and well-being. In the present paper, we aimed to cover this gap in our knowledge.

### 1.5. Aim and Hypotheses of the Present Study

In the present paper, we aimed to investigate the relationships between job insecurity and psychosocial well-being, considering the mediating role played by the feelings of life uncertainty faced by individuals during the recent pandemic period. In particular, we focused on two different aspects of psychosocial well-being, namely job satisfaction and burnout. From this perspective, regarding job satisfaction, we meant the feeling of being happy and satisfied in relation to the work an individual is doing [85], while for burnout, we meant a stress syndrome characterized by emotional and physical exhaustion and interpersonal strain [86]. Job satisfaction and burnout have both been notoriously related to psychological individual and occupational well-being [87]. As previously noted, we intended life uncertainty to be the perception that present and future life is characterized by a sense of precariousness, temporariness, transience, and instability [69]. Eventually, job insecurity was defined as the perceived probability and the fear of losing one’s job position [31,32,58].

**Figure 1:**
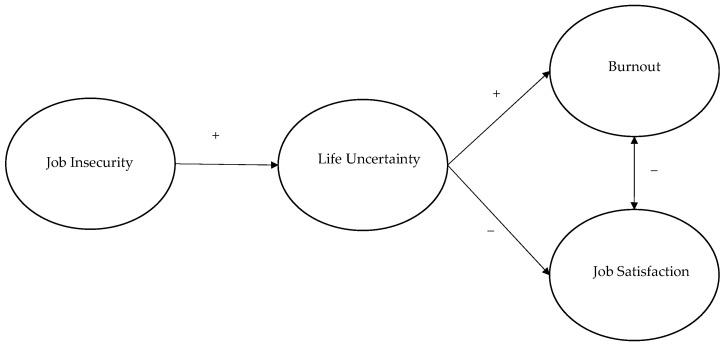
The theoretical model.

We expected that higher job insecurity and higher perceived life uncertainty were related to an impaired psychosocial well-being. Specifically, we anticipated they would be positively related to burnout and negatively related to job satisfaction. More importantly, we expected that the perception of life uncertainty mediated the relationship between job insecurity and both components of psychosocial well-being (see Figure 1 for the theoretical model).

The theoretical rationale of these hypotheses can be described by referring to the transactional theory of stress [33] and the theoretical models of job insecurity [88] that were previously examined in the introduction. As a major stressor, job insecurity fosters negative strain reactions. Therefore, high job insecure individuals should show lower job satisfaction and higher burnout [29,30,31]. Likewise, life uncertainty generally represents both an unpleasant and an uneasy feeling per se [89,90], and a threatening experience for individuals [53,69]. In this line of reasoning, consistent with the transactional theory of stress [33], feelings of uncertainty, instability, and precariousness about what may happen in one’s own life could be regarded to be an important stressor and a vulnerability factor [31,33,34,35,36,37,38,39]. Therefore, life uncertainty was expected to be related to stress reactions affecting psychosocial health, and therefore, lower job satisfaction and higher burnout were expected to be associated with higher life uncertainty.

Chirumbolo et al. [69] investigated the influence of job insecurity and life uncertainty in the realm of consumer behavior, and pointed out how they were jointly related to a reduction in everyday goods consumptions and to the sacrifice of long-term life projects. In the present paper, while building on these previous findings, we expanded our understanding of the phenomena by broadening the empirical horizon and shifting towards the domain of psychosocial well-being. Therefore, here, we proposed that life uncertainty would play a mediating role, suggesting that job insecurity would represent an antecedent of perceived life uncertainty which would be, in turn, be related to a less job satisfaction and more burnout. The present study contributes to our understanding as it represents the first study to empirically investigate the relationships among life uncertainty and well-being related variables and their mediating explanatory roles with respect to job insecurity. In fact, although they could be theoretically grounded in stress theory [33,34,35,36,37,38,39], these predicted relationships have not been previously investigated or demonstrated. In the present paper, therefore, we address this particular lack in our knowledge.

## 2. Method

### 2.1. Participants and Procedures

The data collection for the present study was conducted in Italy, in September 2020, a few months after the national lockdown related to the pandemic health emergency of 2020. Italian workers were invited to complete an online survey and recruited through different social media platforms (for related approach to data gathering see [70,91]). Thus, participants were selected via a non-probabilistic snowballing procedure. Overall, a total of 357 valid questionnaires were collected (M = 146 and F = 211), with an average age of participants of 41.78 y.o. (SD = 13.49). The individual characteristics of participants are reported in Table 1.

Given the online methodological nature of our questionnaire, the response rate was particularly high and about 89% of contacted participants gave their consent to take and complete the survey. Since we ran a web-based survey, in the system settings, it was explicitly required to have an answer for each item. Therefore, the valid questionnaires that we obtained and analyzed did not have any missing values. In view of the high response rate and the lack of missing data in the questionnaire, the problem of “non-response error” was considered to be apparently attenuated. We also aimed to control the effects of social desirability and response set by using bidirectional worded items [92].

Two a priori power analyses were run to establish, in advance, the recommended required sample size to detect a significant bivariate association and to run a structural equation model (SEM) [93]. We expected a small-to-medium effect size of *r* = 0.20, with a power level set at 0.80 and a significant alpha level set at 0.05; therefore, the minimum sample size necessary to detect a significant bivariate association was *n* = 194 [93]. Regarding the SEM, with the same previous parameters, we considered four latent and eleven observed variables, resulting in a required minimum sample size to run a SEM and detect a significant effect of *n* = 342, whereas the minimum sample size required for model structure was *n* = 241 [94]. The sample size of the present study, thus, appeared to be fully satisfactory in terms of statistical power.

### 2.2. Measures

The web-based survey was entirely anonymous and complete privacy assurance was given to participants by the authors. After being notified that the data would be used for solely statistical and scientific goals, participants gave their consent to take the survey, which consisted of a set of questions regarding sociodemographic information and the following measures related to the present investigation.

Job insecurity was assessed using three items re-worded by Chirumbolo et al. [69] which were originally elaborated by Vander Elst and colleagues [95]. This scale assessed the feeling of being unsecure about one’s own job position. An example of an item is “I think I might lose my job in the near future.” Participants answered using a 5-point Likert scale, from (1) completely disagree to (5) completely agree. This scale exhibited good reliability (Cronbach alpha of 0.89).

Life uncertainty was measured with a scale of nine items [69]. This scale measured the perception that the terms of present life are characterized by a sense of precariousness, temporariness, and instability, and that future life projects are framed within a feeling of fragility, vagueness, and uncertainty. An example of an item is “My life is surrounded by a feeling of precariousness.” Participants responded using a 5-point Likert scale from (1) completely disagree to (5) completely agree. This scale showed high internal consistency (Cronbach alpha of 0.93).

Global job satisfaction was assessed using three items from the Job Satisfaction Subscale taken from the Michigan Organizational Assessment Questionnaire [96,97]. These items measured how much an individual was satisfied with own’s one job overall. An example of an item is “All in all, I am satisfied with my job.” Participants rated each item on a 5-point Likert scale, from (1) completely disagree to (5) completely agree, and the scale had a satisfactory internal consistency (Cronbach alpha of 0.75).

The burnout dimension of exhaustion was assessed using three items taken from the Italian Version of the Maslach Burnout Inventory—General Survey (MBI-GS) [98,99]. A sample item is “I feel emotionally exhausted from my work.” The interpersonal strain dimension was measured by means of three items from the Interpersonal Strain at Work scale [100], aimed at measuring the mental and emotional distancing from other people at work. A sample item is “At work I find myself to be insensitive to other people’s problems.” Taken together, these six items measured the burnout dimensions of emotional and physical exhaustion and interpersonal strain. Participants answered each item indicating their agreement/disagreement on a 5-point Likert scale, from (1) completely disagree to (5) completely agree. The six items displayed good internal consistency (Cronbach alpha of 0.84).

Furthermore, a few sociodemographic and contextual variables were also assessed by asking participants to indicate their age and gender, whether they were living with a partner, their level of education and socioeconomic status, the characteristics of their job such as their occupational sector, whether they had a contingent permanent job, whether they had a full-time job, how they perceived the stability/precarity of their job, and whether during the pandemic period they suffered worse economic conditions and worse working conditions.

### 2.3. Data Analyses

The software SPSS V.27 (IBM, Armonk, NY, USA, https://www.spss.it) was used to compute descriptive analyses and correlations among variables. The software M-Plus V.8.3 (https://www.statmodel.com/) was employed to run the structural equation model with latent variables [101].

The present research was approved by the Academic Committee of Sapienza University of Rome (protocol number RM11816433B7B857).

## 3. Results

The correlational analysis pointed out that job insecurity was positively related to life uncertainty and burnout and negatively related to job satisfaction. Life uncertainty was significantly correlated with burnout and negatively correlated with job satisfaction. Burnout was negatively related to job satisfaction. Correlations among variables were all in the hypothesized direction and are reported in Table 2.

Regarding the relationships with sociodemographic and contextual variables (see Table 3), job insecurity was significantly related to lower education, working for a private company, having had experienced worse economic and working conditions during pandemic, having a temporary contingent and precarious employment, and not having a full-time job (Table 3). Life uncertainty was significantly related to living alone, having lower education, having a temporary contingent and precarious employment, not having a full-time job, and having suffered worse economic and working conditions during the pandemic (Table 3). Individuals with higher job satisfaction were those with higher education, living with a partner, with a full-time stable job, who experienced the same or better economic and working conditions during the pandemic period. Burnout was only modestly related to having a full-time job (Table 3).

### 3.1. The Measurement Model

In order to cope with measurement issues, such as possible common method variance effects, a confirmative factor analysis (CFA) was conducted to evaluate whether the measures employed in the present investigation were adequately divergent from each other [102]. Two alternative nested models were compared. In the first model (M1), the fit of a one-factor solution was tested. In the case that the measures were not sufficiently distinct from each other, model M1 would show a satisfactory fit. In the second model, a correlated four-factor solution (M2) was tested, with all variables separated from each other. Afterwards, M2 was formally compared to M1 using a chi-square difference test (Δχ^2^) [103]. Precisely, in the case that our measures would exhibit adequate discriminant validity, it was expected that M2 would show a better fit than M1 in terms of fit indexes. Furthermore, a significant decrease in chi-square from M1 to M2 was to be expected. The results of the CFA pointed out that the one factor solution (M1), i.e., no discriminant validity, exhibited a very poor fit (Table 4). On the contrary, the correlated four-factor model (M2) showed both better fit than the one factor solution (Table 4), and a significantly decrease in the chi-square, Δχ^2^ (6) = 785.89, *p* < 0.001.

**Figure 2:**
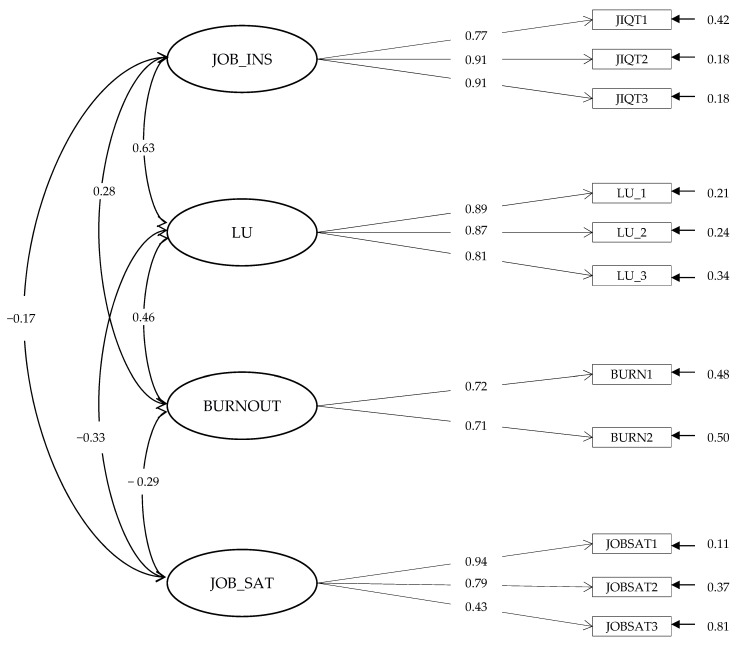
The Measurement Model. JOB_INS, job insecurity; LU, life uncertainty; JOB_SAT, job satisfaction.

The comparison of these two models underlined that our measures were sufficiently distinct from each another and that the four-factor model M2 was to be preferred (see Figure 2).

### 3.2. The Path Model with Latent Variables

We hypothesized a mediation model in which the total effect of job insecurity on job satisfaction and burnout was mediated by the feeling of life uncertainty (Figure 1). To test this model, a mediation analysis with latent variables was performed via SEM, using a partially disaggregated method of analysis [104]. Latent variables were defined using three parcels for life uncertainty and two parcels for burnout [104,105]. The parcels were constructed for each of the latent variables using the item-to-construct balance strategy [105], i.e., parcels were computed to have an equal number of items and comparable reliability. Since job insecurity and job satisfaction were assessed by a limited number of items, their corresponding latent variables were defined using their items as manifest indicators. Therefore, in the final model, a combination of total and partial disaggregation approach was employed [105].

Model fit was evaluated with the following indices: (a) the Comparative Fit Index (CFI), (b) the Tucker–Lewis index (TLI), (c) the root mean squared error of approximation (RMSEA), (d) and the standardized root mean square residual (SRMR). In general, TLI and CFI values that fall between 0.90 and 0.95 are supposed to be good [106,107], while values greater than 0.95 are expected to be very good [107]. Small values of RMSEA and SRMR, on the contrary, suggest a good fit (i.e., values lower than 0.08 [106,107]).

To evaluate the indirect effects, a bootstrapping procedure was used to build the bias corrected 95 percent confidence intervals (95% CI, 5000 samples with replacement) [108,109]. A mediation effect takes place if the indirect effect is statistically significant at *p* < 0.05, something that happens when the zero value does not fall within the 95% CI [108,109].

A two-step mediation strategy was followed [103,110]. Initially, the mediation model was tested, namely the relationships of job insecurity with job satisfaction and burnout were mediated by the feeling of life uncertainty. This full mediation model was named M_mediated_. Secondly, a non-mediated model was examined, in which all the direct effects from job insecurity to job satisfaction and burnout were computed. This model was called M_nonmediated_. The two nested models were contrasted to each other (M_mediated_ vs. M_nonmediated_) and their goodness was evaluated via the chi-square difference test (Δχ^2^) [103]. If this contrast resulted in a non-significant χ^2^, this meant that the mediation model was the model to be retained, since more parsimonious as compared to the non-mediated one.

**Figure 3:**
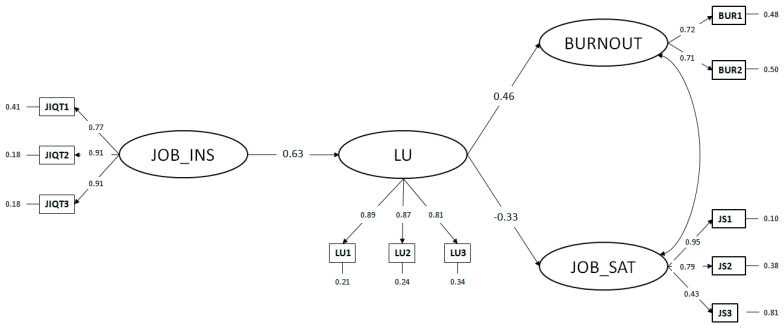
Mediation path analysis with latent variables. Note: Standardized coefficients were reported. All coefficients were statistically significant at *p* < 0.05. JOB_INS, job insecurity; LU, life uncertainty; JOB_SAT, job satisfaction. Fit Indexes: Chi-square (40) = 152.01, *p* < 0.01, CFI = 0.95, TLI = 0.93, RMSEA = 0.08, and SRMR = 0.07.

The analyses showed that the mediation model (M_mediated_) globally exhibited a good fit, chi-square (40) = 152.01, *p* < 0.01, CFI = 0.95, TLI = 0.93, RMSEA = 0.08, SRMR = 0.07. The non-mediated model in which direct effects were computed (M_nonmediated_) appeared to have not increased the model fit, Chi-square (38) = 151.39, *p* < 0.001; CFI = 0.95, TLI = 0.92, RMSEA = 0.09, SRMR = 0.08. The two nested models were then formally compared, and the chi-square difference turned out to be not statistically significant, Δχ^2^ (2) = 0.62, *p* = 0.73. Therefore, the mediation model (M_mediated_) was selected because it was more parsimonious (i.e., it had more degrees of freedom) as compared with the non-mediated model. The statistics of the mediation model are shown in Figure 3. The model highlighted how job insecurity was significantly related to life uncertainty which, in turn, was significantly related to higher burnout and lower job satisfaction. Although the word “effect” may suggest a causal relationship, we can not make inferences about causality due to the cross-sectional nature of the data.

In Table 5, the decomposition of total and specific indirect effects of the mediated model are exhibited. All specific indirect effects from job insecurity to both burnout and job satisfaction mediated by life uncertainty were significant.

## 4. Discussion

The present study investigated the relationships among job insecurity, life uncertainty, and two indicators of psychosocial well-being, namely job satisfaction and burnout. It was shown that both job insecurity and life uncertainty were negatively related to job satisfaction and positively related to burnout, suggesting their global detrimental association with psychosocial well-being. Moreover, the findings of the present investigation suggested that the relationship between job insecurity and psychosocial well-being was an indirect association that occurred though the mediation of life uncertainty.

These findings appear to be relevant, particularly, if framed within the contemporary socioeconomic situation. In fact, the COVID-19 pandemic resulted in a general state of high uncertainty in the social and economic sphere [83,111,112,113,114,115,116]. In the recent literature, a substantial number of studies have broadly documented the labor market outcomes of the pandemic period [13]. Altig et al. [117] analyzed several different indicators of economic uncertainty before and during the COVID-19 pandemic, and showed that all of them highlighted a dramatic rise in uncertainty during the pandemic [117]. Such uncertainty, together with the measures taken during the pandemic such as social distancing and lockdown, were shown to have had severe negative psychosocial, health and socioeconomic effects on many different aspects [13,118] as follows: on labor markets with losses of working hours and jobs [14] and an increase in the unemployment rate [17]; on worsening mental health and well-being [6,7,8,118] with increased anxiety and mood disorders [119,120,121,122] and distress for both parents and children [91]; on employment inequality of minority groups [123,124], and on gender-related outcomes [18,124].

From what previously outlined, it clearly emerges that phenomena such as the pandemic, that entail economic and financial instability, have also increased the general subjective feelings of uncertainty experienced by individuals [4,5,83]. This steady ”state of crisis’— exacerbated by the pandemic environment—has determined several and traverse negative consequences on major economies, health institutions, and policymakers, which represent fundamental pillars with respect to the well-being of individuals and societies. Furthermore, the financial shocks, in a liquid way, have spilled over into the world of work as well. Consequently, unemployment rates have increased dramatically, increasing the perception of job insecurity wherein employees question the continuity about their own job positions. Personal uncertainty and insecurity related to a job are related to evident monetary loss and also affect an individual’s mood and general psychological and emotional health, leading to a general state of uncertainty [4,5] and generalized personal and psychological discomfort [6,7,8].

As other studies have promptly engaged in gathering data on mood disorders and psychological related illnesses affecting workers during the COVID-19 pandemic [6,7,8,91,119,120,121,122], in this paper we focused, instead, on the role of life uncertainty, referring to feelings of existential precarity and uncertainty about one’s life. It seemed very relevant to empirically test this particular variable, since the COVID-19 pandemic has been ongoing for two years now and we are still managing its ramifications. Drawing on the transactional theory of stress [33], in the present paper, we aimed to provide further insight with respect to the relationships among job insecurity and life uncertainty with psychosocial well-being variables (i.e., job satisfaction and burnout) by focusing on the mediating role of life uncertainty. According to the transactional theory of stress [33], when people are exposed to environmental stimuli which are appraised as threatening, and that go beyond an individual’s skill to cope with, a stress reaction can occur [33,34,35,36,37,38,39]. These situational factors, thus, typically produce negative health outcomes [33]. Uncertainty and low predictable situations are generally appraised as stressful leading to stress reactions [33,45]. It has been well documented that the pandemic period has mostly originated an overall increase in uncertainty in many aspects of people’s lives [4,5,11,83,112,114]. In this fashion, our findings largely confirm the proposed mediation framework, in which job insecurity is positively related to workers’ life uncertainty and, in turn, their higher levels of burnout and lower levels of job satisfaction.

Although the detrimental relationships between job insecurity and health outcomes are well established in the scientific literature [4,5,83,111,112,114], the underlying process is still a topic of debate among scholars [29] and current research is more and more focusing on mediators and moderators of job insecurity influence [29,31,63]. The present study aimed to address this question by investigating an overlooked mediator of job insecurity, namely life uncertainty.

In fact, the present results suggest that life uncertainty plays a crucial role to better explain the mechanism that conveys the job insecurity influence on some psychosocial well-being outcomes. Previous studies [69] have investigated the role of life uncertainty in the field of consumer behavior, and showed that life uncertainty was associated with an inclination to reduce individuals’ spending on everyday goods (such as buying foods, drinks, entertainments, and devices) and an increased intention to sacrifice long-term projects (such as getting married, buying a house, and obtaining a loan). In addition, life uncertainty has been shown to play a mediating role in the relationship between job insecurity and these consumer intentions [69]. In the present paper, we shifted the focus to a different domain, namely that of psychosocial health of individuals, by investigating the relationships among life uncertainty, job insecurity, job satisfaction, and burnout. Therefore, the findings of the present study add something new to our knowledge and understanding of the phenomena and the underlying mechanisms, and incrementally contribute to the previous literature showing that the fear of job loss spills over on life uncertainty that negatively affects job satisfaction and burnout. However, there is still a lot to know. In line with the stress perspective [31,33,34,35,36,37,38,39], future studies should persist in this direction by investigating the personal and situational resources that people actually use to cope and face the stress generated by uncertainty in life, focusing more on the moderating variables in the relationship between life uncertainty and health.

As the construct of life uncertainty is particularly suitable with the current historical period, we considered that it was essential to collect the data during the peak of the pandemic. To the best of our knowledge, no other study has previously investigated the relationships among life uncertainty and job satisfaction and burnout during the pandemic period. In the present study, we highlighted life uncertainty as a manifestation of the perception and feeling of precariousness regarding the present and future of one’s own life and we evaluated a model linking job insecurity to job satisfaction and burnout, thus, offering the following contributions to the literature: First, the findings of the model empirically tested indicate that job insecurity is related to life uncertainty in this direction. This, importantly, sheds new light on a relationship that has not been explored in organizational research. Second, this study examines how life uncertainty mediates the relationship between job insecurity and burnout. In examining underlying processes based on workers’ job insecurity and two major psychosocial well-being outcomes, our results indicate that high life uncertainty levels can result in undesirable employee attitudes through its influence. Our findings suggest that life uncertainty is one potential mediating mechanism that explains the relationship of job insecurity with job satisfaction and burnout. Therefore, the present paper provides insights about the importance to also consider life uncertainty in order to investigate the detrimental relationships of job insecurity with well-being outcomes. Furthermore, it may open new research fields, inspiring other authors to explore what personal and contextual factors may affect feelings of life uncertainty.

From a practical point of view, this means that insecure employee, with high levels of the perception and feeling of precariousness regarding the present and future of one’s own life, are negatively associated with job satisfaction and positively related to burnout. Third, our study is the first to explore the mediating mechanisms between a relevant stressor such as job insecurity and job satisfaction and burnout, extending previous research in the literature.

However, this research suffers from some limitation. First, non-probabilistic sampling somewhat limited the generalizability of our results. Participants were selected via a non-probabilistic snowballing procedure, and therefore, the sample could not be considered to be representative of Italian workers. Moreover, this sampling procedure might suffer a lack of representativeness due to selection bias [125]. In Appendix A, the main sociodemographic characteristics of the study sample are compared to those of a representative sample of the Italian working population. Our sample roughly mirrors the core features. However, a few discrepancies in the sample composition can be noted, mainly because, in the study sample, we have more public workers than expected based on a representative sample. This finding suggests that further research on a larger sample, including a greater proportion of workers of the private sector, would be necessary to test the generalizability and robustness of our results. Nevertheless, our power analysis has determined that the sample size was large enough to guarantee more than adequate statistical power. Furthermore, our findings are in line with theoretical predictions and are likewise in line with the results of other research based on representative samples. Considering these aspects, we can be sufficiently confident about the results and conclusions of our study. Secondly, the study employed a cross-sectional study design, which has been widely considered to be inferior to longitudinal research when establishing casual relationships between variables. Furthermore, this design approach is not recognized as the best way to examine mediation effect [126]. Although the use of a longitudinal design approach as a remedy to fix the causality issue is often overstated [127], future longitudinal studies to replicate our results are needed. Third, the self-reported nature of the measures used may represent a limit. As all the measures are based on a self-report survey using one questionnaire, we cannot completely exclude a potential single-method bias. However, all the variables included are highly subjective, and therefore, a self-measure report seems more than appropriate. Moreover, it does not seem plausible that factors such as common-method-variance effects could entirely explain our results. As a matter of fact, the measures employed in the present investigation exhibited adequate discriminant validity to exclude that this effect only would be responsible for the findings [102].

## 5. Conclusions

This study proposed a model to explain the relationships among job insecurity and job satisfaction and burnout that occur through life uncertainty. The results showed that workers’ job insecurity and life uncertainty were both relevant factors that could lower their psychosocial well-being by decreasing their levels of job satisfaction and increasing their burnout and that life uncertainty contributed to mediate and explain these relationships. The three main contributions of these findings include: First, they confirm evidence from previous studies in the literature that workers’ job insecurity is related to job satisfaction and burnout. Second, they shed a new light on a relatively new construct of life uncertainty as a mediator in understanding the job insecurity and two major psychosocial well-being outcome relationships. Third, they empirically suggest the importance of considering the valuable contribution of measuring the precariousness, uncertainty, and temporary nature of one’s present and future life in this historical moment and the role it can play in individuals’ health and their work environment. In conclusion, it appears that both subjective perceptions of job insecurity and life uncertainty are associated with psychosocial well-being, corroborating, at an individual level, what is usually speculated and found at the macro economical and society levels.

## Figures and Tables

**Table 1:** Sociodemographic features of the sample (*n* = 357).

Sociodemographic Characteristics	%
Education level	
1. Middle school	9.7
2. High school	50.7
3. University degree or higher	40.1
Marital status	
1. Single	37.5
2. Married (or lived with a partner)	50.4
3. Divorced	9.4
4. Widowed	2.5
Socioeconomic status	
1. Low	8.4
2. Medium low	24.9
3. Medium	53.5
4. Medium high	12.9
5. High	0.3
Contract	
1. Permanent	51.0
2. Temporary	7.6
3. Self employed	22.4
4. No contract (e.g., occasional job and gig economy)	17.1
Occupational status	
1. Full-time	68.3
2. Part-time	17
3. Occasional	14.6
Productive sector	
1. Industry	21.3
2. Service	76.8
3. Agricultural	2.0
Organizational sector	
1. Public	72.8
2. Private	27.2

**Table 2:** Correlations among the variables with descriptives.

Variables	M	SD	1	2	3	4
1. Job Insecurity	2.31	1.02	1			
2. Life Uncertainty	2.29	0.98	0.59 **	1		
3. Job Satisfaction	3.82	1.04	−0.23 **	−0.32 **	1	
4. Burnout	1.89	0.82	0.18 **	0.36 **	−0.34 **	1

Note. ** *p* < 0.01.

**Table 3:** Correlations with sociodemografic and contextual variables.

	Job Ins	LU	Job Sat	Burnout
Living with a partner	−0.07	−0.18 ***	0.15 **	0.04
Education	−0.11 *	−0.12 *	0.19 ***	−0.01
Occupational sector (private)	0.22 ***	0.04	−0.01	0.07
Socioeconomic status	0.07	0.08	−0.09	0.08
Worse economic conditions	0.38 ***	0.32 ***	−0.14 **	0.01
COVID exposure	0.005	−0.01	0.10	0.03
Contingent job	0.32 ***	0.23 ***	−0.17 **	0.004
Full-time Job	−0.22 ***	−0.22 ***	0.28 ***	0.13 *
Precarity of work	0.47 ***	0.41 ***	−0.27 ***	−0.02
Working conditions during lockdown (worse)	0.33 ***	0.27 ***	−0.19 ***	−0.10

Note: * *p* < 0.05, ** *p* < 0.01, and *** *p* < 0.001. Job Ins, job insecurity; LU, life uncertainty; Job Sat, job satisfaction; living with a partner (0 = no, 1 = yes); education (0 = lower, 1 = higher); occupational sector (0 = public, 1 = private); socioeconomic Status (0 = lower, 1 = higher); worse economic conditions (0 = the same or better, 1 = worse); contingent job (0 = permanent, 1 = temporary); full-time job (0 = no, 1 = yes); precarity of work (0 = stable, 1 = precarious); worse working conditions (0 = the same or better, 1 = worse).

**Table 4:** Confirmative factor analysis of the measurement models.

Models	Chi-Square	df	CFI	TLI	RMSEA	SRMR
M1: One Factor	937.28	44	0.57	0.47	0.24	0.14
M2: Four Factors	151.39	38	0.95	0.92	0.09	0.07

**Table 5:** Specific indirect effects of the mediated model.

Indirect Effect	Effect	SE	*p*	Bootstrap 95% CI
Indirect Effect: JI → LU → Burnout	0.29	0.074	0.01	[0.147; 0.441]
Indirect Effect: JI → LU → Job Satisfaction	−0.23	0.055	0.01	[−0.324; −0.125]

Note. Standardized coefficients were reported. JI = Job Insecurity; LU = Life Uncertainty.

## Data Availability

Data can be provided by the first author upon request.

## Appendix A

**Table A1:** Main sociodemographic features of the sample as compared with a representative sample of the Italian working population in %.

Sociodemographic Characteristics	Study Sample (*n* = 357) %	Representative Sample of Italian Working Population (*n* = 15,076) %
Education level		
1. Middle school	9.7	31.3
2. High school	50.7	42.9
3. University degree or higher	40.1	25.8
Socio-economic status		
1. Low	8.4	8.8
2. Medium low	24.9	23.5
3. Medium	53.5	41.2
4. Medium high	12.9	7.1
5. High	0.3	1.2
Contract		
1. Permanent	51.0	64
2. Temporary	7.6	9.7
3. Self employed	22.4	23.1
4. No contract (e.g., occasional job, gig economy)	17.1	3.3
Occupational status		
1. Full-time	68.3	82.5
2. Part-time	17	17.5
3. Occasional	14.6	NA
Productive sector		
1. Industry	21.3	22.9
2. Service	76.8	73.9
3. Agricultural	2.0	3.2
Organizational sector		
1. Public	72.8	27
2. Private	27.2	73

NA, not applicable. Data from a representative sample of Italian working population were taken from INAPP—ISTITUTO NAZIONALE PER LE ANALISI DELLE POLITICHE PUBBLICHE (National Institute for the Analyses of Public Policies—Italian Ministry of Work and Social Policies) [128].
